# Active-Tuning and Polarization-Independent Absorber and Sensor in the Infrared Region Based on the Phase Change Material of Ge_2_Sb_2_Te_5_ (GST)

**DOI:** 10.1038/s41598-018-30550-2

**Published:** 2018-08-20

**Authors:** Zhongyi Guo, Xiao Yang, Fei Shen, Qingfeng Zhou, Jun Gao, Kai Guo

**Affiliations:** School of Computer and Information, Hefei University of Technology, Hefei, 230009 China

## Abstract

Phase-change materials (PCMs), possessing thermo-optic and thermo-electric properties, have constantly enabled the rewritable optical data storage and the commercialized phase-change memory devices. In particular, Ge_2_Sb_2_Te_5_ (GST) has been considered for configurable photonics applications, such as active dielectric metasurface. In this paper, we report an active absorber with metal-insulator-metal (MIM) scheme with GST in the infrared region. The absorber consists of Al disk and reflective Al film with a GST spacer layer. Extraordinary absorption with peaks of more than 90% can be achieved over a broad bandwidth, attributing to highly confined gap surface plasmon resonance. In addition, the absorption can be tuned via adjusting the proportion of GST crystallization, which is a unique advantage to design active device. Meanwhile, the absorption is polarization-independent owing to its structural symmetry. Furthermore, we introduce the designed absorber to the application of sensing. This nearly perfect absorbing strategy offers great potential in sensing applications due to its flexibility and polarization-independence.

## Introduction

For more than a decade, the field of electromagnetic metamaterials (MMs) has been developing rapidly. By arranging components in subwavelength scale, the emerging artificial material with unnatural electromagnetic properties has been achieved and utilized for numerous applications, such as beam-splitters^1,2^, sensors^3,4^ and perfect absorbers^5,6^. The MMs-based perfect absorbers (MPAs) usually employ a metal-isolator-metal (MIM) construction to achieve impedance matching to incident plane by optimizing its geometrical parameters, thereby forming a resonant dip in the reflectance spectrum. In 2006, Lee *et al*. designed a one-dimensional sandwich structure employing a Fabry-Pérot resonance cavity, obtaining measured reflectance with several sharp reflectance dips and absorption peaks of value close to 90%^7^. In 2008, Landy and Sajuyigbe extended the MIM-based perfect absorber (PA) to two-dimensional case, creating narrow-band in the reflective spectrum by manipulating $\varepsilon$ and $\mu$^8^. In 2016, Chen *et al*. theoretically and experimentally demonstrated an infrared aluminum PA for detecting the C=O vibration mode^9^. Nevertheless, PAs with only one absorption peak cannot satisfy the largely increasing demand of functional devices working over a broadband spectrum.

Although several substantial efforts have been devoted to create multi-reflection dips^10,11^ or broad-band absorption^12,13^, there is a general restriction hindering the application of MPAs in integrated devices. That is non-reconfiguration, because most of the aforementioned MPAs consists of passive meta-atoms. Thus, once the morphology of MMs is specified, its optical response will be determined. For this reason, researchers are now focusing on exploring MMs-based devices with tunable responses by utilizing a number of techniques, such as liquid crystals^14,15^, graphene^16,17^, and phase-change materials (PCMs)^18,19^.

Among them, PCMs is of particular interest since they show a strong dependence of optical properties on applied electrical or thermal stimuli. In general, the main extraordinary property of PCMs is that they can be switched from an amorphous phase to a crystalline phase via thermal, electrical or optical activations. The amorphous-crystalline transition causes a large change in their refractive indexes. There are many phase change materials had been found and researched, such as Ge_1_Cu_2_Te_3_ (GCT), Ge_2_Sb_2_Te_5_ (GST). In comparison with GST, GCT possesses an exceptional amorphous thermal stability, making GCT a suitable candidate for high-temperature PCM applications^20,21^. However, GCT may be more suitable for visible spectrum. In the near infrared (NIR) range, GST alloy has advantages of low optical loss, high stability and quick response to stimuli, in comparison with other PCMs^22^. Furthermore, by virtue of a high crystallization rate and good reversibility between amorphous and crystalline phases, GST has been exploited for kinds of photonics applications, such as multi-level memory, based on GST with different crystallization fractions^23,24^. In addition, in the experiment, we can achieve different intermediate phases of the GST phase-change thin film by applying energy-controlled light sources or managing the different baking times. Although, when structure is completed, we will be in a dilemma to know the crystalline fraction of the GST layer, we can construct the relation between the crystalline fraction and the energy-controlled light sources or different baking times of the GST, in which the crystalline fraction and the effective dielectric constant of the GST can be determined by comparing the simulated spectrum and the experimental spectrum^25^. Therefore, this makes them possible for the apparatus integrated with GST to be applied in practice.

In this paper, we design an active absorber with highly confined gap surface plasmon (GSP) mode supported by MIM structure in the infrared region. The absorber is composed of an Al disk and an Al reflective mirror with a GST spacer layer. Taking the advantage of the switching between amorphous GST (a-GST) and crystalline GST (c-GST) in the designed absorber, we demonstrate the activity of absorber without geometrical variations. Numerical simulations show that this perfect absorption arises from highly confined GSP mode in the Al-GST-Al structure. Further, we have also investigated the polarization sensitivity of the Al-GST-Al structure, which demonstrate polarization-independence characteristics for the absorber. Moreover, this active-tuning and polarization-independent absorbing strategy introduces great opportunities to works for sensor.

## Results and Discussion

**Figure 1:**
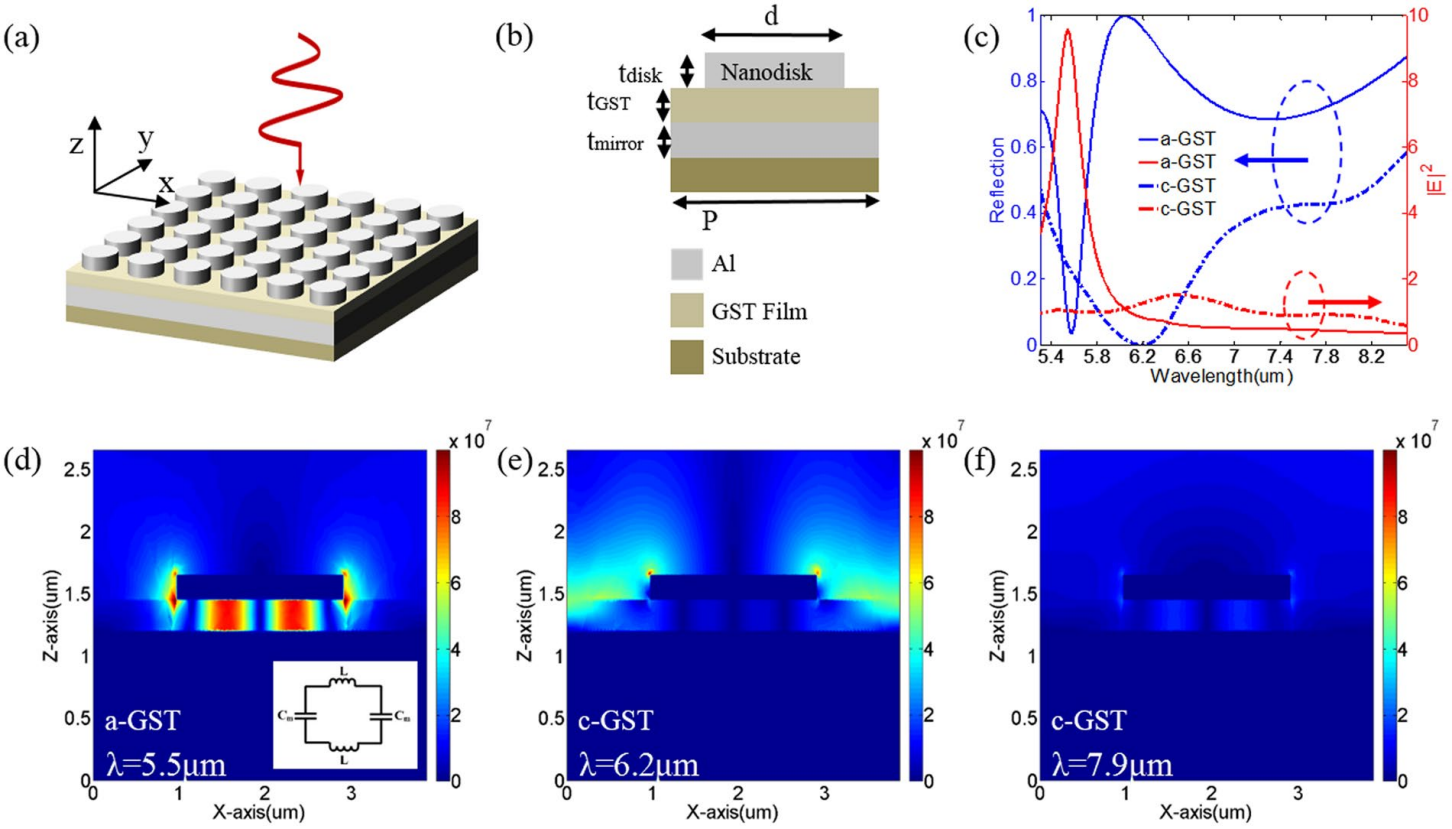
(**a**) Schematic of the proposed structure. (**b**) Cross-sectional view of the proposed structure in unit-cell. The geometrical parameters are set as: P = 3.87 μm, d = 1.93 um, t_disk_ = 0.2 μm, t_GST_ = 0.25 μm, t_mirror_ = 0.1 μm. (**c**) The simulated reflectance spectrum and the enhancement of the near-field distributions intensity with a-GST and c-GST as the dielectric layer in the infrared region. The near-field distributions at (**d**) $\lambda$ = 5.5 μm with a-GST as the dielectric layer and at (**e**) $\lambda$ = 6.2 μm (**f**) $\lambda$ = 7.9 μm with c-GST as the dielectric layer. Inset in (**d**): Equivalent circuit model for the proposed structure under GSP resonance.

Figure 1(a) shows the perspective view of the proposed absorber, which is embedded on transparent silicon substrate and arranged in a two-dimensional square lattice in x-y plane. Figure 1(b) illustrates the cross-sectional view of the absorber’s geometry. P and d are the period of the square lattice and the diameter of Al disk, respectively. t_disk_, t_GST_ and t_mirror_ are the thickness of Al disk, GST spacer, and Al reflective film, respectively. The proposed absorber is illuminated by a plane wave propagating along z-axis. Numerical simulation has been carried out with finite element method (FEM).

The permittivity of Al was taken from the experimental results^26^. The refractive index of silicon substrate is 3.4. For GST, the optical response can be gradually tuned by employing different energies on it, thereby leading to formation of nucleation in a-GST and partial crystalline state^27^. To describe the effective permittivity of GST with various crystallization conditions, Lorentz-Lorenz relation can be employed^28^

1 $$\frac{{\varepsilon }_{{\rm{eff}}}(\lambda )-1}{{\varepsilon }_{{\rm{eff}}}(\lambda )+2}=m\times \frac{{\varepsilon }_{{\rm{cGST}}}(\lambda )-1}{{\varepsilon }_{{\rm{cGST}}}(\lambda )+2}+(1-m)\times \frac{{\varepsilon }_{{\rm{aGST}}}(\lambda )-1}{{\varepsilon }_{{\rm{aGST}}}(\lambda )+2}$$

where ${\varepsilon }_{{\rm{cGST}}}(\lambda )$ and ${\varepsilon }_{{\rm{aGST}}}(\lambda )$ are the wavelength-dependent permittivity of c-GST and a-GST, respectively^29^. *m* is the crystallization fraction of the GST, ranging from 0 to 1^30^.

Firstly, we try to describe the optical property of the proposed absorber. Similar to previous MPAs, the top metal is engineered to achieve impedance matching with the incident medium, giving rise to enhancement of local electric field and nearly perfect absorption^31^. In addition, the bottom reflective metal film should be thick enough to ensure that transmitted light can be negligible. Therefore, the MIM structure could support highly confined gap surface plasmon mode, achieving perfect absorption at the resonant wavelength.

To demonstrate the optical responses of the designed structure, we calculated the reflection and electric field enhancement of the proposed nanostructure with both a-GST and c-GST under the X polarization incidences in infrared region, as presented in Fig. 1(c). The enhancement of the electric field intensity is defined as ${\int }_{V}{|E|}^{2}{\rm{d}}V/{\int }_{V}{|{E}_{0}|}^{2}{\rm{d}}V$, where $V$ is the volume within the designed structure, $|E|$ and $|{E}_{{\rm{0}}}|$ represent the amplitude of the electric field with and without the absorber structure, respectively. In the calculation, the geometric parameters are chosen as: P = 3.87 μm, d = 1.93 μm, t_disk_ = 0.2 μm, t_GST_ = 0.25 μm, t_mirror_ = 0.1 μm. For the a-GST case, there is a reflection dip (R = 0.03) at 5.54 um with near perfect absorption (A = 1 - R - T = 0.97), corresponding to electric field enhancement reaching up to 10 near the wavelength of reflection dip. Note that, there is a small difference in wavelength between the dip of reflection and the peak of electric field enhancement, which is caused by Ohmic loss in the proposed structure. For the c-GST case, the reflection dip (R = 0.43, A = 1 - R - T = 0.57) shifts to 7.9μm owing to the increment of refractive index of GST after phase transition from amorphous to crystalline state. This phenomenon will be explained later.

Figure 1(d,f) are the simulated near field distributions for a-GST case at $\lambda$ = 5.5 μm and c-GST case at $\lambda$ = 7.9 μm, respectively, presenting the typical characteristics of GSP mode. The inset of Fig. 1(d) schematically shows the corresponding equivalent circuit model of GSP mode, where the metal and insulator provide the inductance, L, and capacitance, C, respectively. In this case, the Al disk is capacitively coupled to the Al mirror, altering the effective impedance to achieve a resonance in absorbing electromagnetic waves. Therefore, both the inductance and capacitance change the resonance wavelength $\lambda \propto \sqrt{L{C}_{m}}$. In our design, the capacitance, ${C}_{{\rm{m}}}={\rm{c}}{\varepsilon }_{{\rm{d}}}{\varepsilon }_{{\rm{0}}}d/{t}_{{\rm{GST}}}$, varies with the crystalline fraction of GST layer, where ${\rm{c}}$ is a constant accounting for the non-uniform charge distribution at the metal surfaces, ${\varepsilon }_{{\rm{d}}}$ and ${\varepsilon }_{0}$ are the permittivity of GST and vacuum, respectively. Whereas, the inductance, L, is a constant. It is obvious that with increasing the refractive index of GST, the charge capacity in dielectric structure increases, leading to red-shifting of optical resonances^32^. In addition, Fig. 1(d) shows that most of the electromagnetic field is confined in the a-GST spacer, indicating substantial absorption of incident electric field. In contrast, Fig. 1(f) shows that GSP resonance in c-GST layer are highly loss in GST layer since the Ohmic losses in c-GST are more significant than Al disk in the infrared region^33^. It is in a good agreement with the results in Fig. 1(c) that only 40% of the incident electromagnetic wave is reflected back, and most of the energy is dissipated by the medium and converted into heat, resulting in field enhancement shrinking to 0.92 at $\lambda$ = 7.9 μm. Besides, Fig. 1(e) demonstrates that the absorption of c-GST case at $\lambda$ = 6.2 μm can be attributed to localized surface plasmon (LSP) resonance at the edge of Al disks.

**Figure 2:**
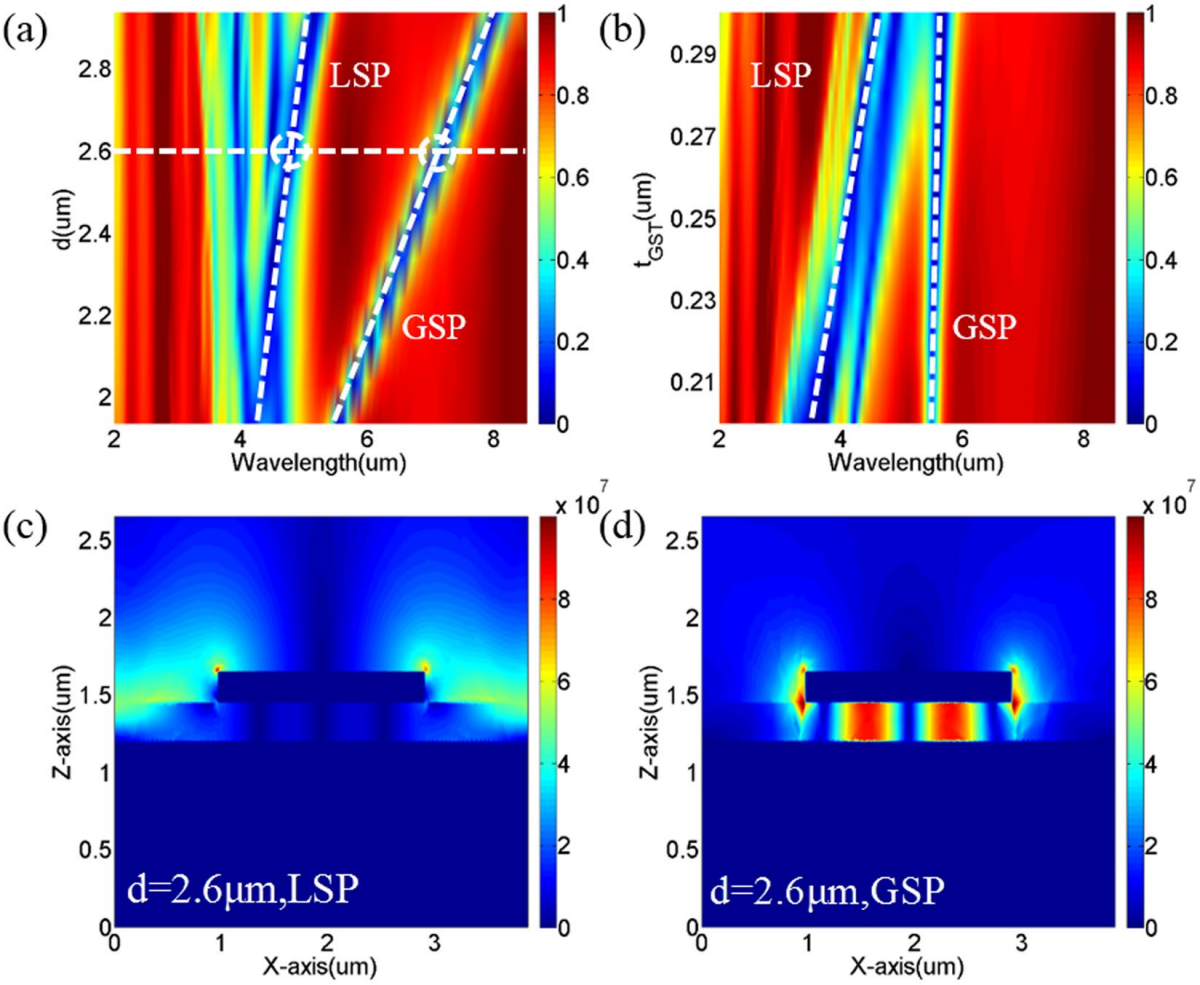
The dependences of reflectance spectrum of the proposed structure with a-GST as the dielectric layer on (**a**) nanodisk diameter d, (**b**) GST thickness tGST. The near-field distributions of (**c**) LSP and (**d**) GSP modes at the points marked in (**a**).

To unveil the physical nature of nearly perfect absorption, it is helpful to calculate the dependence of absorption on the geometry parameters, because surface plasmon modes sensitively depend on structure and composite. According to the above results and surface plasmon theory, the proposed nanostructure with a-GST could support high-quality GSP modes, due to the low intrinsic loss of a-GST in comparison with c-GST. Figure 2(a) shows the reflection spectrum of the proposed nanostructure with a-GST as a function of Al-disk diameter and incident wavelength. The other structural parameters are chosen as: P = 3.87 μm, t_disk_ = 0.2 μm, t_GST_ = 0.25 μm, and t_mirror_ = 0.1 μm. Figure 2(b) shows the dependence of reflection spectrum of the proposed nanostructure with a-GST on the thickness of GST layer. The other parameters are chosen as: P = 3.87 μm, d = 1.93 μm, t_disk_ = 0.2 μm, and t_mirror_ = 0.1 μm. Simulation results show that two branches of plasmon modes are excited in the proposed nanostructure, as indicated by the white-dashed lines. With increasing the Al-disk diameter and GST layer thickness, the resonant wavelengths of both modes red-shift, and meanwhile, the bandgap between them increases accordingly. In addition, these two branches of plasmon modes overlap when d = 1 μm, and only the GSP mode can be excited (not shown here). Figure 2(c,d) show the near-field distributions of the two plasmon modes (as marked in Fig. 2(a)), respectively. From their field distributions, it can be deduced that the branch with high energy originates from LSP mode, while the branch at long wavelength originates from GSP modes. Furthermore, the reflection spectrum confirms that the GSP mode exhibits desired high-quality over a broadband from 6 um to 8 um, when the Al-disk diameter increases from 2 μm to 2.9 μm. These findings strongly suggest to utilize the GSP mode in the proposed nanostructure with low crystalline fraction to achieve an infrared absorber and sensor.

**Figure 3:**
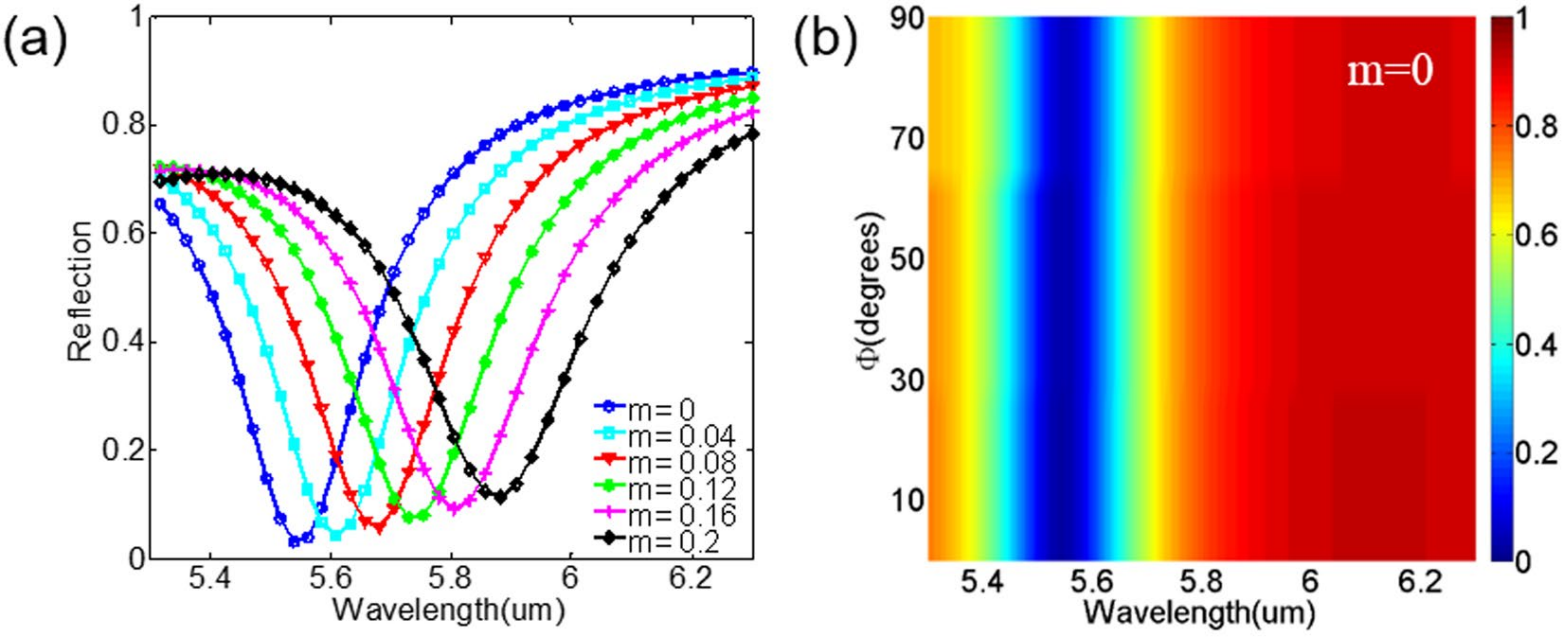
(**a**) The simulated reflectance spectrum of the proposed structure with m = 0, 0.04, 0.08, 0.12, 0.16, and 0.2. (**b**) The simulated polarization-independent of the proposed structure with m = 0.

In fact, one of the key advantages of our device is the utilization of GST layer, which enables us to actively control the pronounced reflection dip in the desired spectrum. Therefore, we are going to discuss absorption controllability of the designed structure. In simulation, we control the state of GST by continuously altering the crystalline fraction (m). Figure 3(a) shows the calculated dependence of reflection on the state of GST with various crystalline fraction of m = 0, 0.04, 0.08, 0.12, 0.16 and 0.20, respectively. The reflection dip corresponding to GSP resonance is red-shifted from 5.55 μm to 5.85 μm, covering a bandwidth of 0.3 μm. The crystalline fraction increases until m = 0.20 because when the crystalline fraction increases, the GSP resonance induced reflection dip becomes wider and shallow, as shown in Fig. 1(c). This effect is determined by the near-field intrinsic loss of GST, which hinders the practical application of such a scheme. When m = 0.20, absorption is still more than 80%, as indicated in Fig. 3(a).

The other advantage of our device is polarization independence owing to the structural symmetry of both unit-cell and lattice. For instance, the reflection spectrum of the designed structure with a-GST is calculated and plotted in Fig. 3(b). The polarization angle ${\rm{\Phi }}$ is rotating from x-direction (0 degree) to y-direction (90 degree). The simulation results manifest the proposed absorber is polarization independent, indicating its great potential in application.

It is feasible for our absorber to work as a sensor, because the sensing principle relies on nearly perfect absorption occurring for a certain surrounding medium. Any variation of the refractive index of surrounding medium may result in reflecting more energy backward (leading to smaller absorption) and resonant wavelength shifting. Therefore, by detecting variations of both the intensity and resonant wavelength in reflection precisely, the sensor for the refractive index can be obtained.

**Figure 4:**
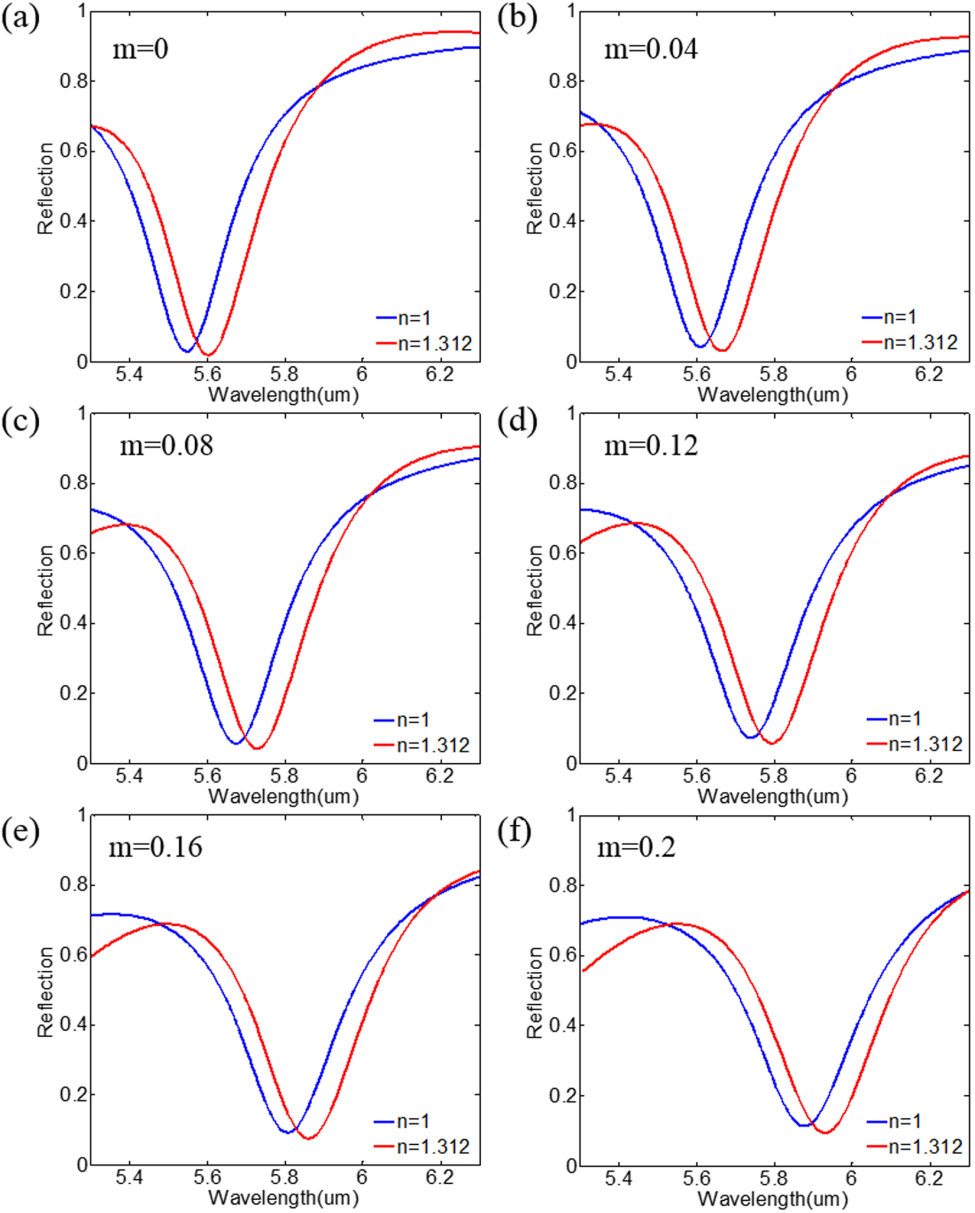
Tuning the simulated reflection spectrum of our designed absorbing sensor for water as a reference, with the control of GST crystalline fraction of (**a**) m = 0, (**b**) 0.04, (**c**) 0.08, (**d**) 0.12, (**e**) 0.16 and (**f**) 0.20. The geometry parameters are chosen as Fig. 1(c). A layer of water with thickness of 0.1 μm is covering on the top of the sensor.

Figure 4 presents the simulated spectra of our designed structure (as absorbing sensor) with different GST crystalline fractions in the spacer layer, when there is a layer of 0.1 μm water covering the absorbing sensor at the top. For comparison, the corresponding cases of air as the covering material are also plotted. It is shown that the working wavelength of refractive index sensing can be actively controlled by tuning the GST phase states. The reflection spectrum of the absorbing sensor maintains an extremely low intensity smaller than 10% for m = 0.2, indicating its ability to work as an efficient sensor. Meanwhile, the sensor exhibits robustness against the crystalline fraction of GST. It is because that for different crystalline states of GST, full width at half maximum (FWHM) and the difference in wavelength of absorption peaks (${\rm{\Delta }}{\rm{\lambda }}$) between the cases of air and water rarely change. In addition, the figure of merit (FOM) that be used to characterize the sensing performance is calculated for the case covering with water by using the expression $FOM=({\rm{\Delta }}\lambda /{\rm{\Delta }}n)/{\rm{FWHM}}$. For m = 0, 0.04, 0.08, 0.12, 0.16 and 0.20, the FOM are calculated to be 0.707, 0.630, 0.616, 0.634, 0.679 and 0.640, respectively. The overall results demonstrate the stable performance of our device working as a sensor.

**Figure 5:**
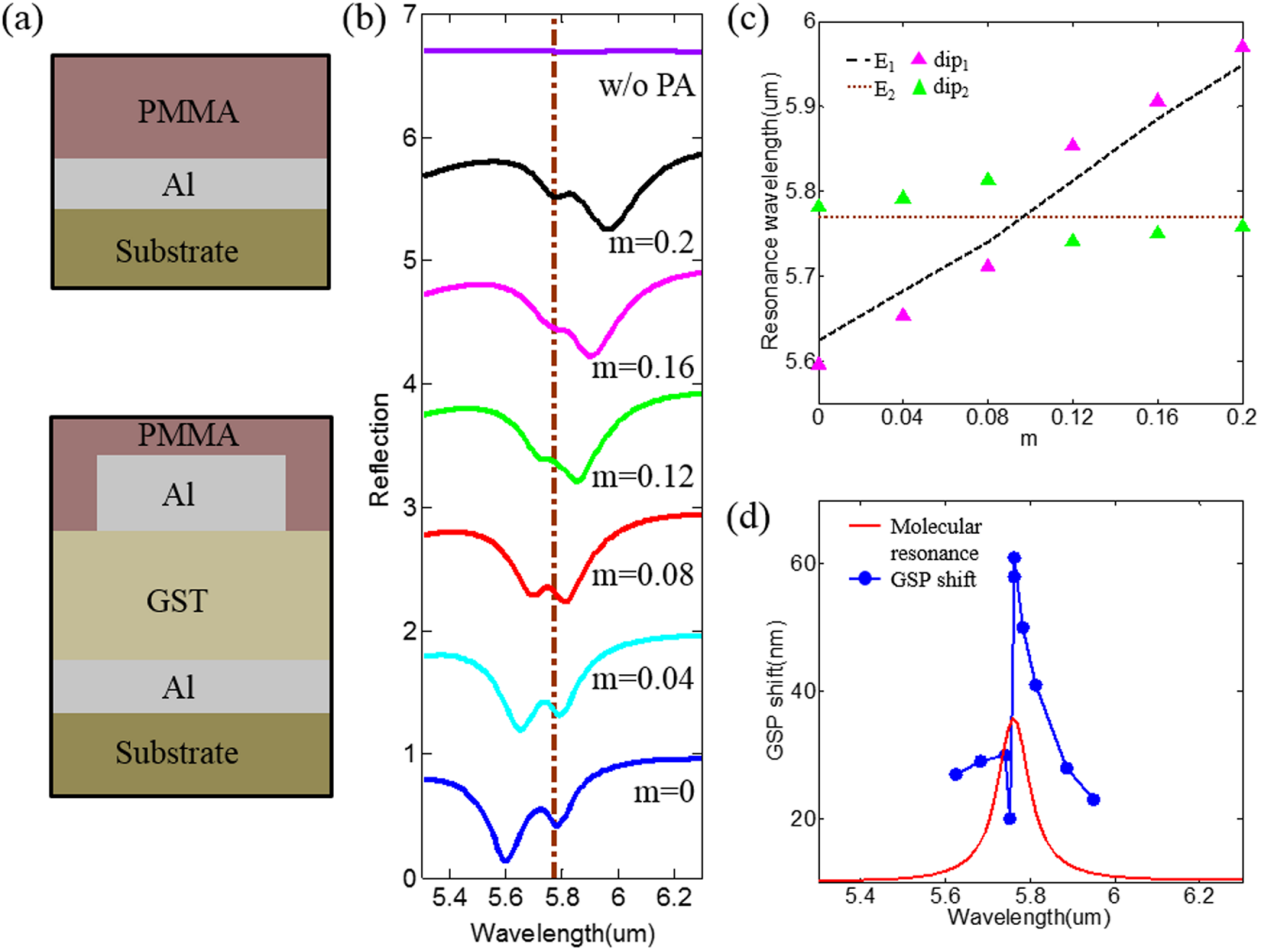
(**a**) Cross sectional schematics of the structures. Top panel: without (w/o) the proposed PA, bottom panel: with the proposed PA. (**b**) Reflectance spectra in different crystalline states of m = 0, 0.04, 0.08, 0.12, 0.16 and 0.2. The dashed line denotes the spectral signature of PMMA carbonyl bond located at 5.76 μm. (**c**) The wavelength of the PA resonance and the vibrational mode were plotted against the crystalline state. (**d**) The GSP shift vs GSP resonance of the proposed PA covered by lossless PMMA (blue points). The red solid line is the molecular vibration spectrum of PMMA.

Furthermore, the working frequency, i.e. the mid-IR range, is particularly well suited for sensing since it covers the range of vibration finger-prints of molecules. Nevertheless, the molecular vibration weakly absorbs the incident field, which makes it difficult to detect the molecules. For example, a PMMA layer with thickness of 200 nm is covering on a thin film of Al and silicon substrate, as shown in top panel of Fig. 5(a). Its reflection spectrum, violet solid line in Fig. 5(b), is relatively smooth and it is hard to observe the spectral signature of PMMA carbonyl bond locating at 5.76 μm. In contrast, when the PMMA layer is covering on our designed structure (as absorbing sensor), the spectral overlapping between PMMA molecular vibration and GSP resonance will lead to increased absorption of PMMA layer around 5.76 μm. Figure 5(b) shows that the overall shape of Fano resonance can be manipulated by tuning the crystalline states of GST, resulting from the revolution of plasmon-molecule interaction.

To better illustrate the revolution of plasmon-molecule interaction, Fig. 5(c) plots the wavelength of reflection dips in Fig. 5(b) as a function of crystalline state of GST, presenting a characteristic mode splitting and anti-crossing. The magenta (dip_1_) and green (dip_2_) triangles denote the absorption of GSP mode and PMMA molecular vibration, respectively, which are confirmed by their near-field distributions (not shown here). The blue dashed line (E_1_) corresponds to the case of lossless PMMA coating, which has only plasmon resonance of PA due to the absence of absorption of PMMA refractive index. The horizontal brown dotted line (E_2_) denotes the wavelength of the PMMA molecular vibration at 5.76 μm. Obviously, plasmon mode strongly interacts with PMMA molecular vibration, forming resonant mode hybridization with the energies of^10,34^

2 $${E}_{\pm }=\frac{{E}_{{\rm{1}}}+{E}_{{\rm{2}}}}{2}\pm \sqrt{\frac{{({E}_{{\rm{1}}}-{E}_{{\rm{2}}})}^{{\rm{2}}}}{4}+{V}_{12}^{2}}$$

where the hybrid “+” and “−” states (*E*_±_) depend on the uncoupled state *E*_1_, *E*_2_, and the coupling strength *V*_12_. Therefore, this hybridization results in the observed characteristic anti-crossing as shown in Fig. 5(c), where the triangles at energies higher and lower than uncoupled energy of ${E}_{{\rm{1}}}$ correspond to hybrid the “+” and “−” states, respectively. In this strong coupling regime, the GSP resonance and molecular vibration cannot be divided into separate components. In addition, the positions of resonances present obvious shift from the two original modes.

Figure 5(d) reveals the influence of the lossy PMMA layer on the GSP resonance shift. The blue dash-dotted line represent the difference in wavelength between GSP resonance of PA with lossy and lossless PMMA layer. The red solid line outlines the absorption spectrum of PMMA for direct comparison. Away from the strong coupling regime, a GSP shift of ~30 nm is observed. When the GSP resonance coincides with the PMMA molecular vibration, the GSP shift drops to ~20 nm owing to the emergence of hybrid modes. When the GSP resonance slightly red-shifts away from the PMMA molecular vibration, the GSP shift recovers and is amplified by 200% over the average value. It is clear that the amplified-plasmon shift due to the strong resonance coupling could benefit the plasmonic sensors. Furthermore, considering the fact that molecular vibrations are typically discrete, these results demonstrate the significant ability of the proposed PA to actively detect various molecules.

To provide a clearer comparison, we summarize the recently reported performance of absorbing sensor in Table 1. Most of them are based on plasmonic structures, in which the working wavelength can be controlled by tuning the geometry parameters. It greatly hinders their practical applications due to its non-tunable ability. Additionally, our device works in the mid-infrared range which is technologically important for a wealth of applications, such as the chemical sensing for the molecular vibration. Based on this table, our proposed device reveals an extraordinary sensing performance beyond different absorbing sensors.

**Table 1:** Comparison between our work and previous reports.

	Active-Tuning	Polarization-Independent	Wavelength Range
Ref.^6^	No	Yes	1.36 μm~2.14 μm
Ref.^35^	No	Yes	0.76 μm~0.96 μm
Ref.^36^	No	Yes	0.60 μm~1.50 μm
Ref.^37^	No	Yes	0.70 μm~1.00 μm
Ref.^38^	No	No	2.39 μm~2.40 μm
**Our Paper**	**Yes**	**Yes**	**5**.**30** **μm**~**6**.**30** **μm**

## Conclusions

In summary, we report an active-tuning and polarization independent absorber in the infrared region based on Al-GST-Al heterostructure, consisting of Al disk and reflective Al film with a GST spacer layer. Extraordinary absorption with peaks of more than 90% can be achieved over a broad bandwidth, attributing to the highly confined GSP resonance. Through adjusting the crystalline fraction, the absorption resonance can be tuned, which is an important advantage for designing the active-tuning absorbers. Meanwhile, we have demonstrated the independence of the proposed absorber on incident polarization owing to its structural symmetry. Moreover, we have also introduced this absorber to work as sensors. Due to its active-tuning flexibility and polarization-independence, our device can be equipped with more practical applications.

## Methods

In our designs, numerical simulation has been carried out with the finite element method (FEM). During the calculations, a plane wave propagating along the z-axis is used as the incidence, where the polarization is along the x-axis and the wavelength range is set in the range of 2 μm~8.5 μm. Meanwhile, perfectly matched layers (PMLs) have been adopted at both top and bottom boundaries in z-direction to avoid nonphysical reflections of outgoing electromagnetic waves. In addition, periodic boundaries have been used in both x and y-directions to save calculation memory and time.
